# Telephone Monitoring of Isolated Patients With Suspected COVID-19 Disease in Primary Care: Prospective Cohort Study

**DOI:** 10.3389/ijph.2022.1604747

**Published:** 2022-08-30

**Authors:** Valle Coronado-Vázquez, Elena Benito-Alonso, Marina Holgado-Juan, Maria Silvia Dorado-Rabaneda, Cristina Bronchalo-González, Juan Gómez-Salgado

**Affiliations:** ^1^ B21-20R Group, Instituto Aragonés de Investigaciones Sanitarias, Universidad de Zaragoza, Zaragoza, Spain; ^2^ Las Cortes Health Centre, Madrid Health Service, Madrid, Spain; ^3^ Universidad Francisco de Vitoria, Madrid, España; ^4^ El Viso de San Juan Health Centre, Servicio de Salud de Castilla La Mancha, Toledo, Spain; ^5^ Illescas Health Centre, Toledo, Spain; ^6^ Yuncos Health Centre, Servicio de Salud de Castilla La Mancha, Toledo, Spain; ^7^ Sociology, Social Work and Public Health, University of Huelva, Huelva, Spain; ^8^ Safety and Health Postgraduate Programme, Escuela de Posgrado, Universidad de Especialidades Espíritu Santo, Guayaquil, Ecuador

**Keywords:** public health, COVID-19, SARS COV-2, primary care, coronavirus disease, comorbidities, telemedicine

## Abstract

**Objective:** Isolation of suspected cases of COVID-19 has been shown effective in reducing disease transmission and monitoring these patients from primary care allows to detect complications. The objective of this study is to determine the evolution of a cohort of patients with suspected COVID-19, and to analyse the factors associated with hospital admissions due to their unfavourable evolution.

**Methods:** Prospective cohort study. A cohort of 166 patients with COVID-19 symptoms was selected and was followed-up by telephone calls during 14 days of home isolation.

**Results:** By the end of the follow-up, a hospital admission had taken place in 14.7% of patients. The mean survival time until admission among diabetics was 12.6, 10.9 days for chronic kidney diseases, and 9.3 days in immunocompromised patients. Immunosuppression was a risk factor for admission over 50 years of age.

**Conclusion:** Hospital admissions for suspected cases of COVID-19 are associated with diabetes, chronic kidney disease, and immunosuppression. Telephone monitoring of these patients from primary care allows for home isolation and early detection of disease complications.

## Introduction

The SARS-CoV-2 (Severe Acute Respiratory Syndrome Coronavirus-2) pandemic has had a rapid expansion in Spain from mid-March [1], at which point, health services have been reorganised to deal with the high number of 2019 novel coronavirus disease (COVID-19) cases that have been occurring daily, in a context of scarce material and human resources [2].

With regard to primary care, the centres have gone from caring for chronic patients in person, to mainly providing telephone care to suspected and confirmed cases of COVID-19. The goal during the pandemic peak, not having availability for community diagnostic testing, has been the detection of suspected cases, their isolation, and follow-up until the appearance of complications of the disease or the disappearance of symptoms [3].

The most common symptoms of COVID-19 are fever, cough, and fatigue, although headache, diarrhoea, and dyspnoea may also occur, among others [4, 5]. Mild cases have been reported that only require supportive measures, but in certain population groups such as the elderly and people with chronic diseases the infection can be serious and even fatal [5].

According to epidemiological criteria, “any person with a clinical picture of sudden onset of acute respiratory infection or any severe state, among others, with fever, cough, or feeling short of breath, is defined as a suspicious case. Other symptoms such as odynophagia, anosmia, ageusia, muscle aches, diarrhoea, chest pain, or headache may also appear” [6].

Most of the studies have been conducted in the hospital environment, describing an increase in the rate of fatal cases in patients with comorbidities such as cardiovascular disease, diabetes, chronic respiratory diseases, hypertension, and cancer [7–9], but usually these risks are not adjusted by factors such as age and sex.

The use of health communication technologies, such as videoconferencing or e-mail, has spread in recent years, promoting patients’ access to health services, reducing transfers to hospitals, and enabling medical consultations from patients’ homes [10]. Although its usefulness to maintain medical care in emergencies has already been demonstrated in the case of natural disasters [11], the COVID-19 pandemic has accelerated the implementation of telemedicine with the use of video calls, phone calls, and emails for communication with patients who stay isolated at home [12]. Since its implementation to reduce transfers to emergency services or the possibility of maintaining telematic consultations by quarantined health professionals, its objective has been, in all cases, to limit transmission of the virus [13–15].

The high risk of contagion in health facilities, both for patients and professionals, and the high pressure on primary care during the period of increased community transmission of the virus [16], has made home monitoring in cases of mild disease desirable, changing the concept of “patient-centred medicine” to “community-centred medicine” [17].

In this context, it is considered that studying the home follow-up of patients with symptoms of COVID-19, carried out by family doctors, can provide knowledge about the evolution of the disease and the advantages of telemedicine for the management of these patients during their isolation.

## Methods

### Objectives

The objectives of this study are:

• To determine the evolution of a cohort of patients with suspected COVID-19 disease, who have been followed-up by primary care consultations through phone calls.
• To analyse the factors associated with hospital admission regarding unfavourable disease developments.

### Study Design and Participants

A prospective cohort study was conducted. Study participants were selected from 23 March to 20 May 2020 by consecutive sampling among all patients over the age of 18 who had consistent symptoms of SARS-CoV-2 infection (fever, cough, dyspnoea, tiredness, odynophagia, headache, arthromyalgia, anosmia, or dysgeusia, diarrhoea, or anorexia). These patients were isolated at home for 14 days.

The sample size for an expected healing ratio of 75%, a relative risk of 0.78, an alpha risk of 5%, and a power of 90%, adding 20% for losses, was 160 patients. “Healing” or “clinical improvement” and “hospital admission” were described as final events. “Healing” or “clinical improvement” was considered when the total or partial disappearance of symptoms occurred, and “hospital admission” when the discharge diagnosis was SARS-CoV-2 infection.

### Data Collection

During the sample recruiting period, an important portion of the population had difficulties in accessing a physician, governments developed systems to filter the calls to medical centres, and only very affected people achieved to be attended by telephone.

In the first months since the start of the SARS-CoV-2 pandemic, serological tests and PCR (Polymerase Chain Reaction) tests were not available in primary care to detect the disease. Family doctors detected suspected cases from the presence of COVID-19-compatible symptoms, which were isolated at their homes with the aim of reducing community transmission.

Each patient joined the study on the first day he or she contacted the family doctor, manifesting the presence of symptoms of the disease. At that time, a telephone follow-up was initiated from primary care with appointments every 48 h, which ended at 14 days or earlier if healing or hospital admission occurred. These patients could develop symptoms from several days before the start of follow-up. During this period, they were asked about the clinical evolution and the appearance of new symptoms, requiring attending the consultation in case of suspicion of a severe evolution of the disease.

Data on sociodemographic variables, history of contact with a SARS-CoV-2 infected person, symptom types, date of onset of symptoms, comorbidities, result at the end of follow-up, and time until the final event occurred were collected.

The data were collected during the interview with the patient and from the medical history by family doctors of five different consultations belonging to the health area of Illescas (Toledo), following a standardised format.

### Statistical Analysis

Whether the patient with symptoms of COVID-19 disease had clinical improvement or healing was assessed, or if a hospital admission was required was studied. The time until the final event occurred, and the influence of comorbidity was observed. The Student’s T-test was used to compare the continuous variables and the Chi-squared test was used for the categorical ones. Survival techniques were used using the Kaplan-Meier method, considering the time until clinical healing or improvement, or hospital admission. Multiple regression was performed using the Cox’s proportional risk model to estimate the hazard ratio and 95% CI for each factor after adjusting by age and sex.

For the processing of the data, the SPSS version 24 was used.

### Ethical Aspects

This study has been conducted in accordance with the protocol and standards of good clinical practice on human research, as described in the Helsinki Declaration. Written informed consent has been requested from all participants prior to their inclusion in the study. Researchers guarantee the confidentiality of data related to the study participants.

The study obtained the positive evaluation of the Clinical Research with Medicines Ethics Committee of the Hospital Complex of Toledo (Spain).

## Results

A cohort of 166 adult patients with one or more symptoms of COVID-19 disease was selected. The sociodemographic and clinical characteristics of patients are presented in Table 1. 50.6% of patients with COVID-19 symptoms were between 41 and 66 years old, and 56.5% of those admitted to the hospital were over 66 years of age (*X* ^2^ = 30.7; *p* < 0.0001).

**TABLE 1:** Patients’ characteristics (COVID-19 Atención Primaria Project, Spain, 2020).

	All patients	Healing or clinical improvement	Hospital admission
Sex *n* (%)			
Female	89 (53.9)	81 (58.7)	6 (25.0)
Male	76 (46.1)	57 (41.3)	18 (75.0)
Age (years) (SD)	49.5 (16.9)	46.6 (15.6)	66.2 (14.9)
<40 years	52 (31.7)	51 (37.0)	1 (4.3)
41–66 years	83 (50.6)	72 (52.2)	9 (39.1)
>66 years	29 (17.7)	15 (10.9)	13 (56.5)
Comorbidities *n* (%)			
Hypertension	42 (25.5)	29 (21.0)	13 (54.2)
Diabetes	11 (6.6)	5 (3.6)	6 (25.0)
COPD	12 (7.2)	9 (6.5)	2 (8.3)
Dyslipidaemia	10 (6.0)	8 (5.8)	2 (8.3)
Cerebrovascular disease	8 (4.8)	5 (3.6)	3 (12.5)
Cardiovascular disease	6 (3.6)	5 (3.6)	1 (4.2)
Cancer	3 (1.8)	2 (1.4)	0 (0)
Chronic kidney disease	8 (4.8)	4 (2.9)	4 (16.7)
Immunosuppression	3 (1.8)	1 (0.7)	2 (8.3)
Contact with patient COVID-19 cases *n* (%)	79 (48.2)	67 (48.6)	11 (47.8)
Symptoms *n* (%)			
Fever	74 (44.6)	62 (44.6)	12 (50)
Cough	83 (50.0)	70 (50.4)	12 (50)
Tiredness	44 (26.5)	39 (28.1)	5 (20.8)
Dyspnoea	20 (12.0)	15 (10.8)	5 (20.8)
Odynophagia	13 (7.8)	11 (7.9)	2 (8.3)
Headache	23 (13.9)	20 (14.4)	3 (12.5)
Arthromyalgia	14 (8.4)	11 (7.9)	3 (12.5)
Anosmia or dysgeusia	9 (5.4)	8 (5.8)	1 (4.2)
Diarrhoea	31 (18.7)	27 (19.4)	4 (16.7)
Anorexia	9 (5.4)	6 (4, 3)	3 (12.5)

SD, standard deviation; COPD, chronic obstructive pulmonary disease.

38% of patients had any morbid pathology, being 70.8% in those who were admitted (*X* ^2^ = 13.4; *p* < 0.0001). The most common comorbidity was high blood pressure (25.5%), followed by Chronic Obstructive Pulmonary Disease (COPD) (7.2%). 48.2% of participants reported having had close contact with patients diagnosed with SARS-COV-2 infection. The most common symptom was coughing (50%), followed by fever (44.6%) and tiredness (26.5%) (Table 1).

At the end of the follow-up, there had been clinical healing or improvement in 85.3% of patients, and hospital admission in 14.7%.

The mean time from the onset of the symptoms until the clinical diagnosis was 4.6 days (SD: 3.7). On average, the time from the onset of the symptoms until healing or improvement was 13.8 days (Range from 3 to 33), and until hospital admission, it was 10.2 days (Range from 2 to 24) (*p* = 0.002).

Compared to those who were healed or had a clinical improvement, there were more hospital admissions at an older age (t-student = 5.58; *p* < 0.001), among males (*X* ^2^ = 9.33; *p* = 0.002), those with hypertension (*X* ^2^ = 11.7; *p* = 0.001), diabetes (*X* ^2^ = 14.8; *p* < 0.001), chronic kidney disease (*X* ^2^ = 8.33; *p* = 0.004), and immunosuppression (*X* ^2^ = 6.56; *p* = 0.01). There was no meaningful association with the number or types of symptoms present in the diagnosis, nor with the number of comorbidities.

**FIGURE 1:**
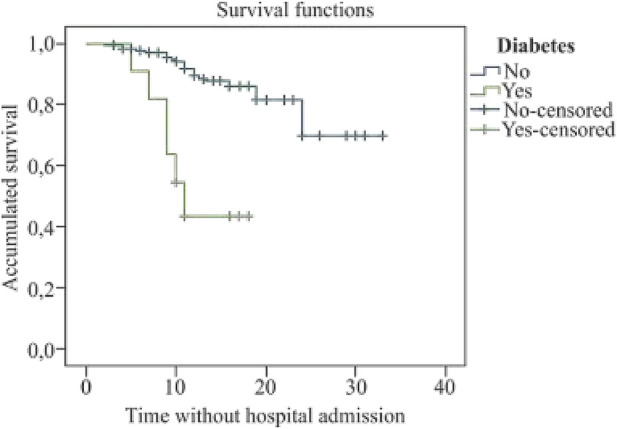
Kaplan Meier’s survival curve for diabetes (COVID-19 Atención Primaria Project, Spain, 2020).

**FIGURE 2:**
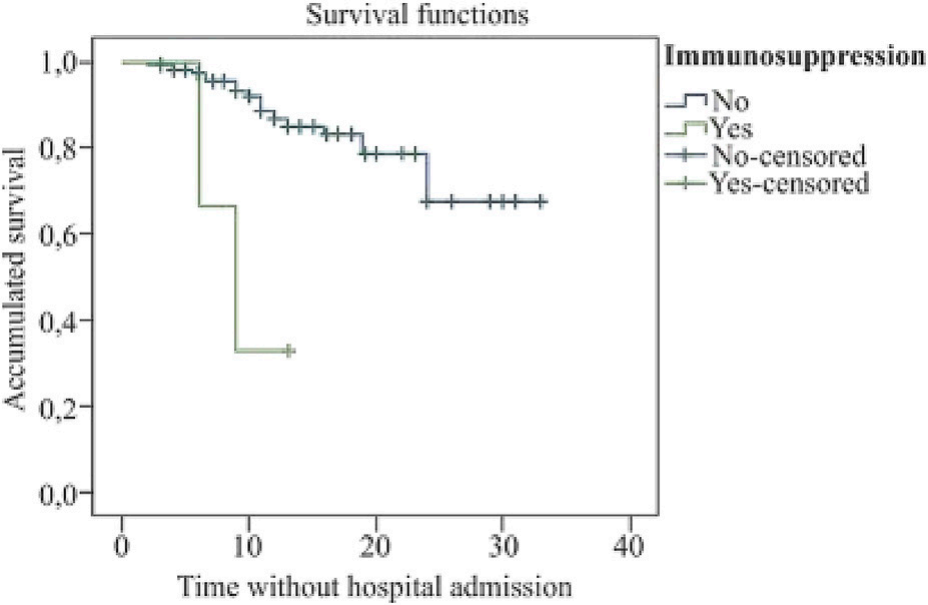
Kaplan Meier’s survival curve for immunosuppression (COVID-19 Atención Primaria Project, Spain, 2020).

**FIGURE 3:**
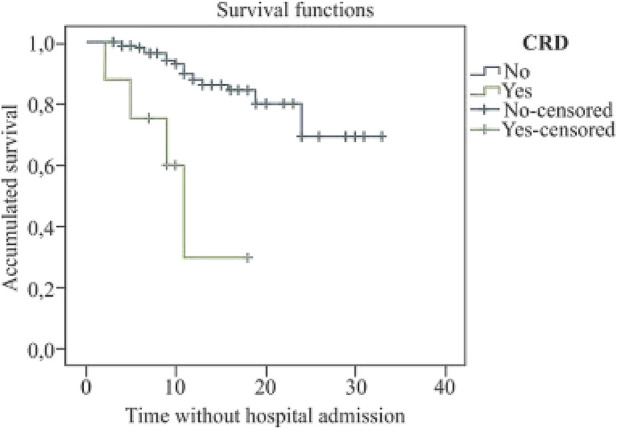
Kaplan Meier’s survival curve for chronic respiratory disease (COVID-19 Atención Primaria Project, Spain, 2020).

The mean survival time until hospital admission in patients with diabetes was 12.6 days (95% CI: 9.7–15.6) (*X* ^2^ = 19.6; *p* < 0.001); in patients with chronic respiratory disease, it was 10.9 days (95% CI: 6.4–15.3) (*X* ^2^ = 18.4; *p* < 0.001), and of 9.3 days in immunosuppressed patients (95% CI: 6.0–12.5) (*X* ^2^ = 11.4; *p* = 0.001) (see Figures 1–3).

The risk of hospital admission multiplied by 5.3 in males, by 9.7 in patients over 50 years of age, by 3.9 in hypertensive, 6.3 in diabetics, 7.6 in those with chronic kidney disease, and by 8.2 in those immunosuppressed (Table 2).

**TABLE 2:** Risk of hospital admission by age, sex, and comorbidity (COVID-19 Atención Primaria Project, Spain, 2020).

	Hazard ratio (95% CI)	*p* value
Hypertension	3.93 (1.75–8.82)	0.001
Diabetes	6.38 (2.48–16.45)	0.0001
COPD	1.26 (0.29–5.39)	0.755
Dyslipidaemia	1.66 (0.38–7.17)	0.491
Cerebrovascular disease	2.74 (0.81–9.27)	0.105
Cardiovascular disease	1.25 (0.16–9.32)	0.825
Chronic respiratory disease	7.66 (2.54–23.08)	0.0001
Immunosuppression	8.24 (1.90–35.75)	0.005
Sex	5.33 (2.07–13.71)	0.001
Age (older than 50 years)	9.71 (2.87–32.78)	0.0001

COPD: chronic obstructive pulmonary disease.

In patients over the age of 50, as compared to those who are younger, having immunosuppression is a risk factor for hospital admission. Among this group, there are no significant differences in the risk of admission for patients with hypertension, diabetes, or chronic respiratory disease. In males, as compared to females, having diabetes, chronic kidney disease, or immunosuppression significantly increases the risk of admission (Table 3).

**TABLE 3:** Effect of comorbidity on hospital admissions, adjusted by age and sex (COVID-19 Atención Primaria Project, Spain, 2020).

	Hazard ratio (95% CI)	*p* value
Age, older than 50		
Hypertension	1.12 (0.39–3.21)	0.821
Diabetes	2.76 (0.88–8.67)	0.081
Chronic respiratory disease	3.16 (0.95–10.47)	0.060
Immunosuppression	6.04 (1.16–31.37)	0.032
Sex (male)		
Hypertension	1.83 (0.67–4.95)	0.23
Diabetes	3.15 (1.02–9.65)	0.04
Chronic respiratory disease	6.93 (2.09–23.01)	0.002
Immunosuppression	20.17 (3.4–119.5)	0.001

## Discussion

The main contribution of this study is that it presents the results of telephone follow-up for 14 days of patients with suspected COVID-19, carried out from primary care. In this cohort, hospital admission is independently associated with diabetes, chronic kidney disease, or immunosuppression.

This paper has highlighted the importance of telemedicine as an alternative to face-to-face consultations to maintain continuity of care in patients isolated on suspicion of COVID-19. Other studies also conclude that telephone consultations in primary care are useful for the practice of care and may be considered similar to face-to-face consultations for health promotion activities, triage, and long-term management of chronic diseases [10, 18]. Telephone consultations during the pandemic have allowed close contact with patients with mild symptoms to early detect complications of the disease, maintaining isolation and thus reducing the risk of transmission of the virus.

Most of the patients who were admitted to hospital were male and elderly. These data are consistent with those found in other studies, although Chen et al. [19] or Li et al. [20], on a sample of people with pneumonia, the median age was less than that described in our work.

Less than half of the patients reported having had contact with people diagnosed with SARS-CoV-2 infection. This percentage is low compared to the study by Wu et al. [7], which described exposure of up to 86% of cases. It is likely that the lack of diagnostic means during the peak of the pandemic in Spain has contributed to this percentage being lower than that detected in studies in countries where diagnostic tests were more available.

Most patients developed fever, cough, tiredness, and diarrhoea, being dyspnoea most common among those requiring hospital admission. The frequency of the onset of symptoms is similar to all published case series [5, 9, 21, 22], indicating stability in the clinical manifestations of the infection.

In this cohort, followed-up by primary care, the percentage of hospital admissions is low as compared to that in other studies [5], where it can rise to 68%. This may be due to the relatively young sample of patients, where more than 82% of the sample were under 66 years of age.

The time from the onset of the symptoms to hospital admission was shorter than in those patients who were healed or had a good clinical evolution, indicating that complications of the disease are more common in the first weeks [20]. The mean time from the onset of the symptoms until patients contacted healthcare professionals has been 5 days, as in other studies [21], which translates the difficulties that have existed in the first months of the epidemic to early detect suspicious cases and isolate them at home.

In our study, only 38% of the sample had some comorbidity, the most common being hypertension and COPD in the total sample, and hypertension, diabetes, and chronic kidney disease in the subgroup of those admitted to hospital. The risk of hospital admission increased, after adjusting by age and sex, in those who had diabetes, chronic kidney disease, or immunosuppression. Several studies [8, 23, 24] associate diabetes with a poorer prognosis of COVID-19 disease. However, in the case of chronic kidney disease, there are few studies that present an increased risk of fatal events [25, 26].

No association has been found between the presence of cardiovascular disease and hospital admissions, although this has been frequently described in other studies [9, 25], which may be due to the mean age of patients not being very high.

Unlike the study by Xu et al [21], in our cohort, there is no higher risk of admission associated with the number of comorbidities.

Knowing the factors associated with an unfavourable evolution of COVID-19 is relevant for primary care, in that it allows preventive measures to be implemented earlier in patients with certain comorbidities, especially if they are elderly.

The strengths of this study relate to the fact that it is the first prospective cohort of patients with suspected COVID-19 disease followed-up by primary care through telephone contact, reflecting the importance of the use of communication technologies during this pandemic.

This study also has some limitations. There have been three losses during the follow-up, from patients who did not respond after several phone calls and could not be located by other means. Although the diagnostic criteria for suspicious case have been followed, as it is a very little-known disease, some cases with atypical symptomatology may not have been detected. With no diagnostic tests, confirming the disease was only possible in the patients who were admitted to hospital.

### Conclusion

The telephone monitoring from primary care of suspected COVID-19 cases isolated at home has allowed to maintain continuity of care, detecting those patients who required a hospital admission by an unfavourable evolution of the disease. Since it is associated with age and prior comorbidities, it would be desirable to design the preventive measures to be taken to reduce the occurrence of fatal events from primary care.

The knowledge that primary care professionals have of their patients and the possibility of close monitoring of symptoms by telephone or with the use of other communication technologies is important to decrease the transmission of the disease by maintaining the isolation of those affected, as well as early detecting the most serious cases requiring hospital treatment.

## Abbreviations

CI, confidence interval; COPD, chronic obstructive pulmonary disease; COVID-19, 2019 novel coronavirus disease; PCR, polymerase chain reaction; SARS-CoV-2, Severe Acute Respiratory Syndrome Coronavirus-2; SD, standard deviation.
